# Insect Eggs Can Enhance Wound Response in Plants: A Study System of Tomato *Solanum lycopersicum* L. and *Helicoverpa zea* Boddie

**DOI:** 10.1371/journal.pone.0037420

**Published:** 2012-05-15

**Authors:** Jinwon Kim, John F. Tooker, Dawn S. Luthe, Consuelo M. De Moraes, Gary W. Felton

**Affiliations:** 1 Department of Entomology, Pennsylvania State University, University Park, Pennsylvania, United States of America; 2 Department of Soil and Crop Science, Pennsylvania State University, University Park, Pennsylvania, United States of America; 3 Center for Chemical Ecology, Pennsylvania State University, University Park, Pennsylvania, United States of America; AgroParisTech, France

## Abstract

Insect oviposition on plants frequently precedes herbivory. Accumulating evidence indicates that plants recognize insect oviposition and elicit direct or indirect defenses to reduce the pressure of future herbivory. Most of the oviposition-triggered plant defenses described thus far remove eggs or keep them away from the host plant or their desirable feeding sites. Here, we report induction of antiherbivore defense by insect oviposition which targets newly hatched larvae, not the eggs, in the system of tomato *Solanum lycopersicum* L., and tomato fruitworm moth *Helicoverpa zea* Boddie. When tomato plants were oviposited by *H. zea* moths, *pin2*, a highly inducible gene encoding protease inhibitor2, which is a representative defense protein against herbivorous arthropods, was expressed at significantly higher level at the oviposition site than surrounding tissues, and expression decreased with distance away from the site of oviposition. Moreover, more eggs resulted in higher *pin2* expression in leaves, and both fertilized and unfertilized eggs induced *pin2* expression. Notably, when quantified daily following deposition of eggs, *pin2* expression at the oviposition site was highest just before the emergence of larvae. Furthermore, *H. zea* oviposition primed the wound-induced increase of *pin2* transcription and a burst of jasmonic acid (JA); tomato plants previously exposed to *H. zea* oviposition showed significantly stronger induction of *pin2* and higher production of JA upon subsequent simulated herbivory than without oviposition. Our results suggest that tomato plants recognize *H. zea* oviposition as a signal of impending future herbivory and induce defenses to prepare for this herbivory by newly hatched neonate larvae.

## Introduction

Upon herbivory, plants induce a variety of defenses that developed via coevolution with herbivorous arthropods, especially insects [1]–[3]. With intensive study during the past few decades, it is now generally understood that upon insect herbivory plants perceive insect-derived cues (e.g. continuous feeding damage, herbivore-associated molecular patterns or HAMPs) and initiate a set of defenses tailored to given herbivore species [3]–[6]. Compared to constitutive defenses, which are continuously expressed irrespective of herbivory, induced defenses are considered more flexible and efficient [7], [8].

Recently, increasing research interest has focused on the deployment of plant defense traits prior to herbivory [9], [10]. The basic premise is that early-induced defenses could be even more effective and adaptive than defenses induced after herbivores start feeding. By perceiving reliable cues of impending herbivory and initiating appropriate defenses in advance, plants may be able to totally avoid or significantly reduce herbivory even before a full-induced defense is activated [9], [10]. Thus far, plants appear to recognize at least three events as indicators of future herbivory. First, some plants increase resistance against insects when a neighboring plant suffers insect herbivory [11], [12]. In this case, plants appear to “eavesdrop” on volatile organic compounds released by the neighboring plant under herbivory and elicit their defenses. Moreover, the volatile-receiving plants showed priming of defenses, meaning the receiver plants activated faster or stronger defenses upon the anticipated herbivory [12]–[16]. Second, insect footsteps can induce defensive responses in plants either by caterpillars breaking cells when crochettes dig into leaves [17] or when caterpillars or moths break trichomes [18]. Third, oviposition, one of the most common events preceding insect larval herbivory, can induce a variety of direct and indirect defenses of plants [19], [20]. Mechanisms of oviposition-induced defenses include production of ovicides [21], a hypersensitive response or necrosis leading to drying or dropping of eggs [22]–[24], excessive growth of hard tissue (neoplasm) under the eggs to force neonates to hatch outside and be exposed to harsh environment [24], [25], egg crushing [26], egg extrusion [27], and calling in egg or larval parasitoids by the host plant [28]–[30].

While most of previous studies of oviposition-induced plant defense have focused on defenses that remove or kill insect eggs from the host [19], [20], there have been only two reports of the effect of insect oviposition on the quality of the host plant as food source and thus on the performance of emerging neonates. *Pieris brassicae* L. oviposition on *Arabidopsis thaliana* L. appeared to suppress antiherbivore defenses and the application of *P. brassicae* egg extract resulted in improved growth of *Spodoptera littolalis* larvae on the host plant [31]. More recently, preceding oviposition treatment with pine sawfly (*Diprion pini* L.) was shown to reduce the performance of the conspecifics on Scots pine (*Pinus sylvestris* L.) branches, although pine sawfly oviposition on pine needles involves mechanical damage by ovipositors and deposition of eggs inside the wound [32].

**Figure 1:**
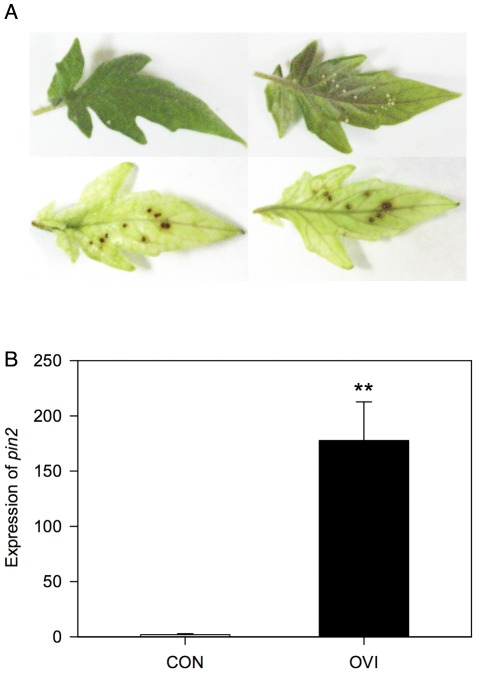
Response of tomato leaves to *H. zea* eggs at the oviposition site. (A) H_2_O_2_ production under eggs of *H. zea* was visualized by DAB staining on an oviposition-treated tomato leaf. *Left panels*, the upper surface of a leaf; *right panels*, the lower surface of a leaf; *upper panels*, before DAB staining; *down panels*, after DAB staining. (B) Induction of *pin2* expression at the *H. zea* oviposition site. Relative *pin2* expression is presented in the graph. Data were analyzed for significance with non-parametric Proc GLM (Mean ± SE; ** above bars indicate significant difference; Chi-square = 6.8182, *p* = 0.009, *n* = 5).

In this study, we hypothesized that tomato plants recognize *H. zea* oviposition as an indicator of future herbivory and induce or prime defenses targeting neonates to hatch. To test the hypothesis, we first investigated whether tomato plants reacted to *H. zea* oviposition and elicited defensive responses at the oviposition site. We examined hydrogen peroxide (H_2_O_2_) production on tomato leaves under *H. zea* eggs, as reactive oxygen species including H_2_O_2_ are often related to antiherbivore plant defenses [33], [34]. Then, we measured the transcriptional level of *pin2*, a gene encoding protease inhibitor2 (Pin2), at the oviposition site, assessed the effect of *H. zea* oviposition on the induction of *pin2* at the oviposition site, and determined the spatial and temporal dynamics of *pin2* expression pattern. The level of *pin2* expression was selected as a defense index because the induction of *pin2* by mechanical wounding and arthropod herbivory is well understood in tomato [6], [35], [36] and because Pin2 is a defensive protein that targets insects under active feeding, not eggs. We also tested whether *H. zea* oviposition primed antiherbivore defense of tomato plants, i.e. whether oviposition-treated tomato showed intensified defense induction upon subsequent herbivory by measuring *pin2* expression and jasmonic acid (JA) concentration in tomato leaves. JA is a plant hormone that orchestrates the induction of antiherbivore defenses [37], and its concentrations in leaves are a good marker of the plant defense level and were successfully used to indicate priming in a previous report [9].

## Results

### Tomato perceives *H. zea* oviposition and induces defensive responses at the oviposition site

#### *Helicoverpa zea* oviposition elicits H_2_O_2_ accumulation at the oviposition site on tomato foliage

It has been proposed that H_2_O_2_ plays a role as a second messenger between early response genes (e.g. genes involved in the biosynthesis of JA) and late response genes (e.g. genes whose products function as defensive traits such as protease inhibitors) [33], [36]. In addition, accumulation of H_2_O_2_ and other reactive oxygen species at the oviposition site was previously reported [34]. Production of H_2_O_2_ at the oviposition site was also detected in the interaction between tomato and *H. zea*. When *H. zea* egg-laden tomato leaves were stained with 3,3′-diaminobenzidine (DAB) solution, H_2_O_2_ production was clearly visualized right under the eggs (Figure 1A).

#### *Pin2* is expressed at the *H. zea* oviposition site and the level of expression decreased with distance from the egg

**Figure 2:**
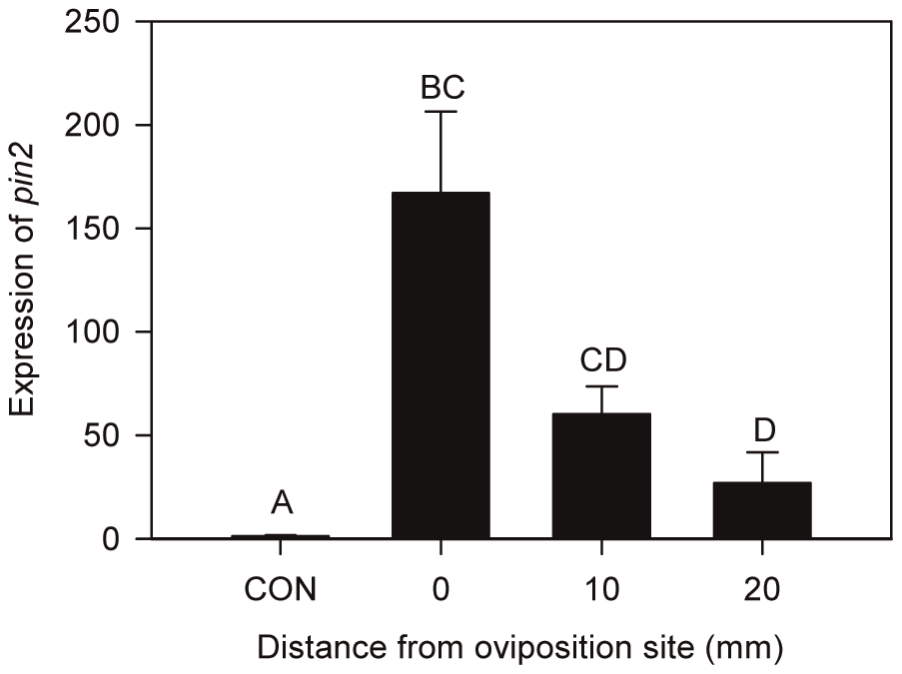
Intensity of *pin2* induction with distance from eggs. Relative *pin2* expression is presented in the graph. Data were analyzed for significance with non-parametric Proc GLM and compared with Tukey test (Mean ± SE; letters above bars indicate significant difference; Chi-square = 14.4695, *p* = 0.0023, *n* = 4 or 5).

Leaf tissue sampled at the *H. zea* oviposition site showed significantly higher level of *pin2* expression (Figure 1B; Non-parametric GLM; Chi-square = 6.8182, *p* = 0.009, *n* = 5). The area of *pin2* expression was more extensive than expected. Transcriptional levels of *pin2* at 0, 10, and 20-mm away from eggs were significantly higher than that of intact plants, and the intensity decreased with distance from (Figure 2; Non-parametric GLM; Chi-square = 14.4695, *p* = 0.0023, *n* = 4 or 5).

#### *Pin2* expression at the oviposition site was highest just before the emergence of neonates

**Figure 3:**
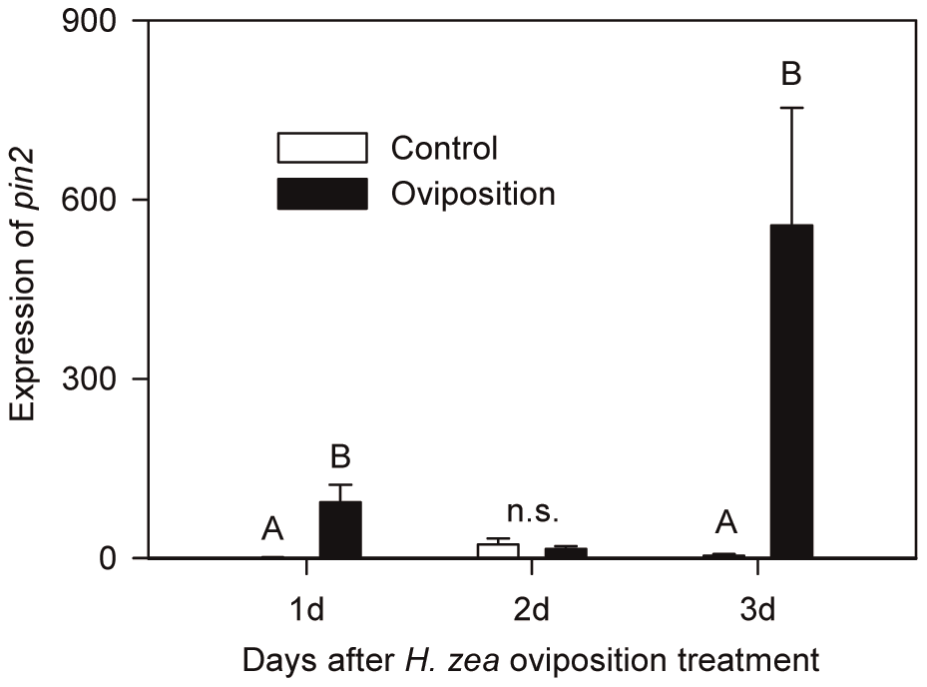
Temporal fluctuation of transcriptional level of tomato *pin2* at the *H. zea* oviposition site. Relative *pin2* expression is presented in the graph. Data were collected for 3 days from the oviposition treatment to the emergence of neonates and analyzed for significance with non-parametric Proc GLM (Mean ± SE; letters above bars indicate significant difference; Day 1, Chi-Square = 3.9382, *p* = 0.0472, *n* = 5; Day 2, Chi-Square = 0.2400, *p* = 0.6242, *n* = 4 or 5; Day 3, Chi-Square = 6.0000, *p* = 0.0143, *n* = 4 or 5).

To understand its temporal dynamics following oviposition, we tracked levels of *pin2* expression at the oviposition site over three days after oviposition and before the emergence of neonates (Figure 3). *Pin2* expression after 1d was significantly higher at the oviposition site than that of intact plants (Non-parametric GLM; Chi-Square = 3.9382, *p* = 0.0472, *n* = 5). There was no difference in levels of *pin2* expression between the two groups on day 2 (Non-parametric GLM; Chi-Square = 0.2400, *p* = 0.6242, *n* = 4 or 5). However, levels of *pin2* expression dramatically increased on day 3 (Non-parametric GLM; Chi-Square = 6.0000, *p* = 0.0143, *n* = 4 or 5), the final day before the emergence of neonates.

#### Unfertilized eggs induced *pin2* as well

**Figure 4:**
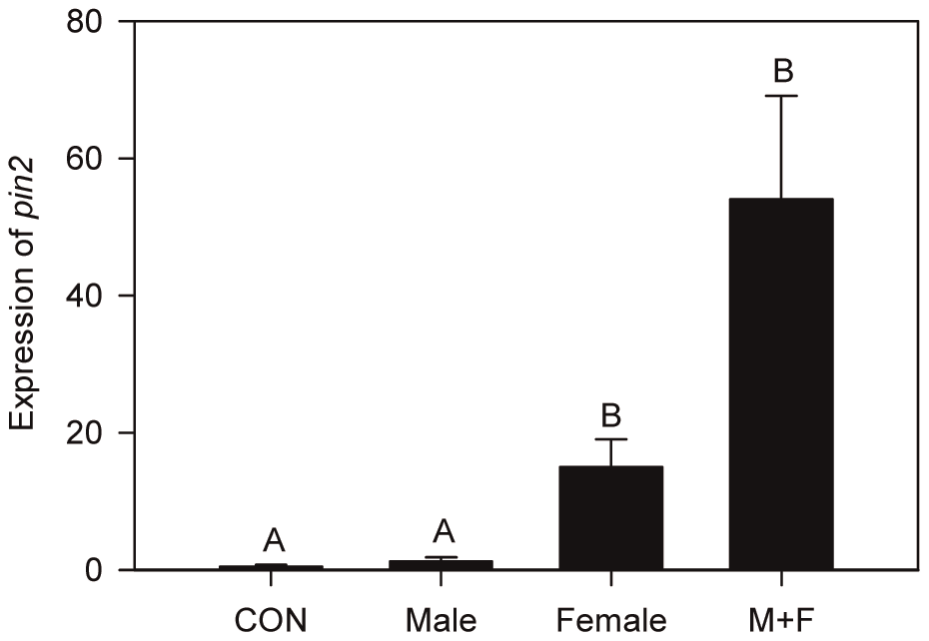
Effect of the egg fertility on tomato *pin2* expression at the *H. zea* position site. Relative *pin2* expression is presented in the graph. Data were analyzed for significance with Proc GLM and compared with Tukey test (Mean ± SE; letters above bars indicate significant difference; *F*_3,15_ = 22.99, *p*<0.0001, *n* = 4 or 5).

A considerable portion of females of many insect species fail to mate in the field [38]. Many female moths of *H. zea* caged with males were found unmated, but they laid about half as many unfertilized eggs as fertilized eggs deposited by mated females [39]. As only fertilized eggs produce neonates and result in herbivory, we examined whether tomato plants would respond to infertile eggs as well as fertile ones. In this experiment, plants were caged with no moth, with male moths only, with virgin female moths only, and with male and female moths together. From now on, we will refer to the female moths that were caged with male moths as ‘mated’ female moths whether they are virgin or mated, although not all females in the group of ‘mated female moths’ are mated. Virgin female moths laid seemingly as many eggs on tomato plants as mated females. Infertility of the eggs laid by virgin female moths was confirmed as the eggs desiccated on tomato leaves in a few days, while caterpillars hatched from the eggs from mated female moths. No significant transcriptional difference in *pin2* was observed between intact plants and plants caged with male moths only. The eggs from mated female moths induced *pin2*, consistent with the results stated above. Interestingly, significant induction of *pin2* was elicited at the oviposition site of unfertilized eggs. Although the mean of *pin2* expression of tomato leaf tissue under unfertilized eggs appeared lower than that of fertilized ones, the difference was not statistically significant (Figure 4; Proc GLM; *F*_3,15_ = 22.99, *p*<0.0001, *n* = 4 or 5).

### Induction of *pin2* and accumulation of JA were primed by *H. zea* oviposition for subsequent simulated *H. zea* herbivory

**Figure 5:**
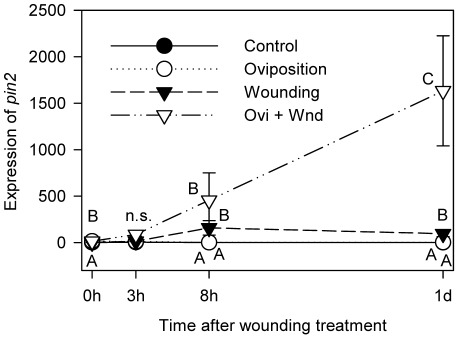
Priming effect of H. zea oviposition on tomato *pin2* expression. Effect of previous *H. zea* oviposition on the induction of tomato *pin2* upon following simulated herbivory was investigated. *Control*, intact plants without oviposition treatment (closed circle); *Oviposition*, plants treated only with oviposition (open circle); *Wounding*, plants mechanically damaged and OS-applied without oviposition treatment (closed triangle); *Ovi+Wnd*, plants treated with oviposition followed by mechanical wounding and OS application (open triangle). Without mechanical damage, there are only *Control* and *Oviposition* at time *0 h*. At times *8 h* and *1 d*, closed circles (control) are hidden behind open circles (oviposition). Relative *pin2* expression is presented in the graph. Data were analyzed for significance with non-parametric Proc GLM and compared with Tukey test (Mean ± SE; letters next to spots indicate significant difference; n.s., data not significantly different; at 0 h, Chi-Square = 21.00, *p* = 0.0025, *n* = 4; at 3 h, Chi-Square = 6.3240, *p* = 0.0969, *n* = 4 or 5; at 8 h, Chi-Square = 13.2857, *n* = 5, *p* = 0.0024; at 1 d, Chi-Square = 14.3843, *n* = 4 or 5, *p* = 0.0024).

Our results thus far strongly suggest that tomato plants perceive *H. zea* eggs and elicit a defensive response. We further hypothesized that *H. zea* oviposition may prime antiherbivore defenses of tomato in anticipation of herbivory by neonates hatching from eggs. To test this hypothesis, we exposed tomato plants to egg-laying *H. zea* moths, and then mechanically wounded the terminal leaflet and applied fresh oral secretion (OS; a mixture of regurgitant and saliva) of *H*. *zea* larvae to simulate insect herbivory. Compared to the typical pattern of *pin2* expression, which increases and then decreases within 24 hr after wounding, tomato plants previously exposed to *H. zea* oviposition showed much stronger induction of *pin2* following mechanical wounding (Figure 5; Non-parametric GLM; at 0 h, Chi-Square = 21.00, *p* = 0.0025, *n* = 4; at 3 h, Chi-Square = 6.3240, *p* = 0.0969, *n* = 4 or 5; at 8 h, Chi-Square = 13.2857, *n* = 5, *p* = 0.0024; at 1 d, Chi-Square = 14.3843, *n* = 4 or 5, *p* = 0.0024). Simple disruption of glandular trichomes, which had been recently reported to induce *pin2* expression [18] did not prime *pin2* expression (Figure S1).

**Figure 6:**
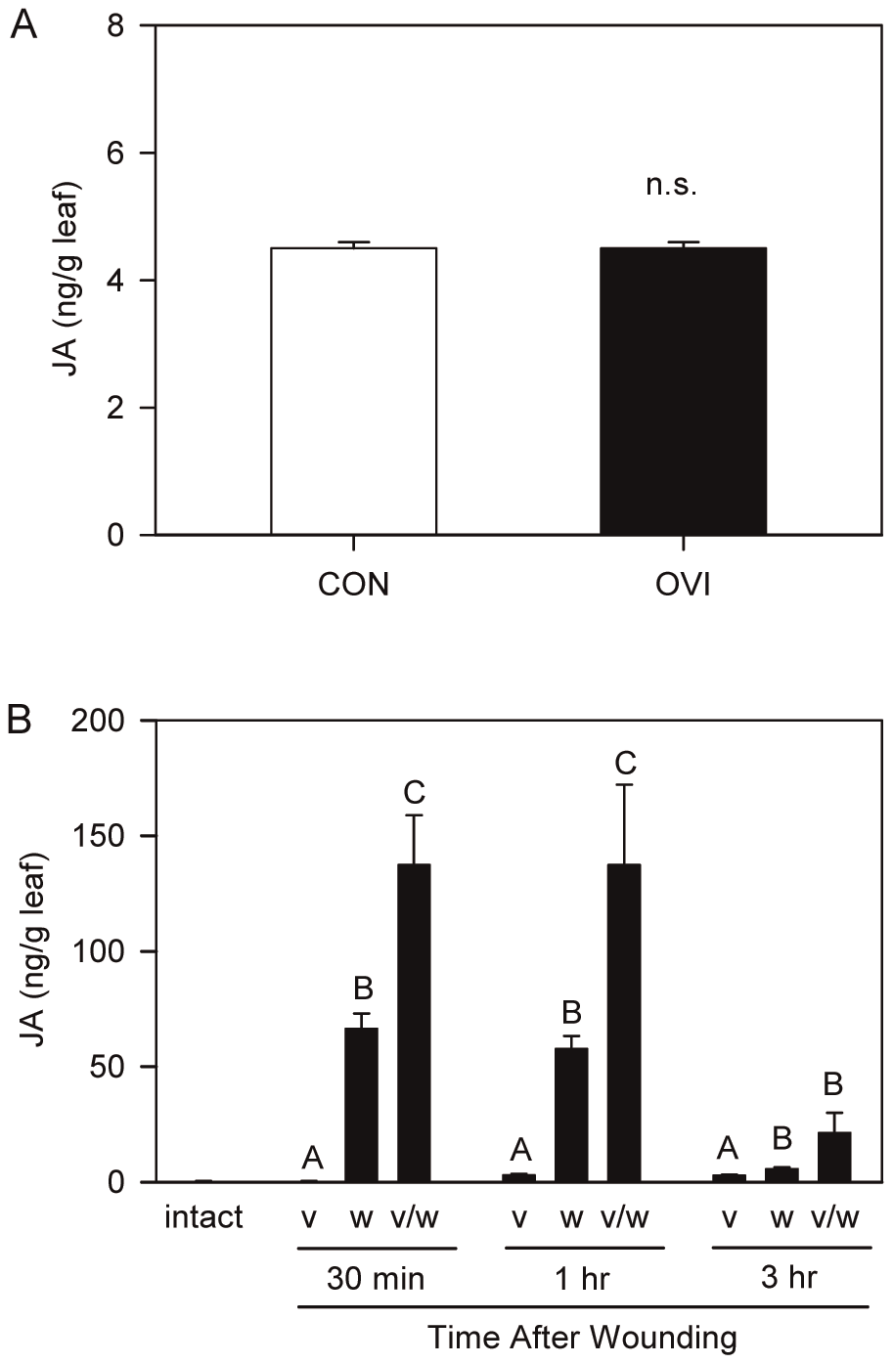
Priming effect of *H. zea* oviposition on JA levels in tomato leaves. (A) Effect of *H. zea* oviposition on basal JA levels. Data were analyzed for significance with Proc GLM (Mean ± SE; n.s., data not significantly different; Proc GLM; *F*_1,8_ = 0.03, *p* = 0.8600, *n* = 5). (B) Effect of previous *H. zea* oviposition on the induction of JA production by mechanical wounding and application of *H. zea* OS. Data were analyzed for significance with non-parametric Proc GLM and compared with Tukey test (Mean ± SE; letters above bars indicate significant difference; n.s., data not significantly different; at 30 min, Chi-Square = 11.2604, *p* = 0.0036, *n* = 5; at 1 hr, Chi-Square = 11.18, *p* = 0.0037, *n* = 5; at 3 hr, Chi-Square = 9.7582, *p* = 0.0076, *n* = 4 or 5). Abbreviations: *v*, tomato plants treated with *H. zea* oviposition; *w*, tomato plants treated with mechanical wounding and application of *H. zea* OS; *v/w*, tomato plants treated with *H. zea* oviposition followed by mechanical wounding and application of *H. zea* OS.

In addition to gene expression data, we also investigated the influence of insect oviposition on JA production after simulated herbivory. We found that oviposition did not change the basal JA levels in leaf tissue (Figure 6A; Proc GLM; *F*_1,8_ = 0.03, *p* = 0.8600, *n* = 5). However, when plants were mechanically wounded and treated with OS of *H. zea* 5^th^ instars to simulate herbivory, JA levels were significantly higher in oviposition-treated plants than in intact plants (Figure 6B; Non-parametric Proc GLM; at 30 min, Chi-Square = 11.2604, *p* = 0.0036, *n* = 5; at 1 hr, Chi-Square = 11.18, *p* = 0.0037, *n* = 5; at 3 hr, Chi-Square = 9.7582, *p* = 0.0076, *n* = 4 or 5). Enhanced level of *pin2* expression and JA burst strongly indicate that tomato defenses are primed by *H. zea* oviposition.

## Discussion

Our results are consistent with the hypothesis that host plants can perceive cues associated with oviposition and then induce and prime defensive responses that can beeffective against soon-to-emerge neonates. The response of tomato to *H. zea* oviposition was comprehensively explored using a suite of defensive responses that are reliable indicators of tomato defense against feeding by insect herbivores. As Pin2, the end product of *pin2*, acts as a defensive trait only after ingested into the insect digestive system, induction or priming of *pin2* by insect oviposition suggests tomato plants recognized the eggs as a future danger and became prepared for herbivory by the neonates, not the eggs. It was demonstrated that the local production of H_2_O_2_ under *H. zea* eggs, the coincidence between the distribution of eggs and transcriptional map of *pin2* on tomato leaves, and the coincidence between the time of the highest *pin2* expression and the larval hatching time. All of these results indicate that the induction of tomato defense by *H. zea* oviposition was caused not by other factors such as trichome disruption, but by the eggs.

There have been two reports showing that insect oviposition can influence the quality of the host plant as food source [31], [32]. The eggs of *P. brassicae* accumulated salicylic acid, a plant hormone acting antagonistically against JA [37], at the oviposition site and suppressed antiherbivore defenses in *A. thaliana* [31]. As a result, *Spodoptera littoralis* Boidsduval caterpillars (but not *P. brassicae* larvae) performed better on the *A. thaliana* previously treated with the extract of *P. brassicae* eggs [31]. More recently, oviposition by pine sawfly adults on the Scots pine was shown to reduce the performance of the conspecific larvae, although the relevant defense mechanism of the host plant was not elucidated [32]. In the present study, we showed that insect oviposition can induce defenses that are known to inhibit the growth of feeding insects and that plant defenses can be primed by insect oviposition. Besides egg deposition, there are other factors associated with oviposition that may induce defensive responses from tomato. For example, disruption of glandular trichomes by moth walking on leaves has been found to induce tomato *pin2* [18]. However, the focused nature of our results just around the oviposition site strongly suggests that cues associated with egg deposition or the egg itself are at least the primary factor that tomato plants perceive to trigger defense induction.

Hydrogen peroxide molecules were clearly visualized under eggs laid singly on leaf surface (Figure 1A). Hydrogen peroxide and other reactive oxygen species function as key cellular signaling molecules [40], and in tomato H_2_O_2_ has been demonstrated to mediate early defense response genes (e.g. genes involved in JA biosynthesis) and late defense response genes such as *pin2* [33], [36]. Hydrogen peroxide production has also been detected beneath eggs of the specialist lepidopteran *P. brassicae* on leaves of *A. thaliana* [34], although in this case, H_2_O_2_ production appears a part of elicitation of hypersensitive response and suppression of plant antiherbivore defense by insect oviposition [31]. Another recent paper documented H2O2 production, JA and JA-regulated wound responses in tomato by the oviposition of *Orius laevigatus* [41]. *Orius* oviposition accompanies mechanical damage because its eggs are not laid, but thrusted into leaf tissue, which probably elicited wound response in tomato.

The expression of *pin2* was found upregulated in a broad area on leaves around the egg deposition site, including 20-mm away (Figure 2). The induced area was large enough that eggs laid a few centimeters apart would activate *pin2* transcription in a whole tomato leaflet. The non-uniform expression of *pin2* on leaves that was highest at the oviposition site might be important as a defense trait. After emerging, neonates wander searching for suitable feeding sites within or between host plants [42]. Neonates of *H. zea* will hatch where *pin2* expression is highest and move away to find more desirable feeding sites. Because predation is one of the main mortality factors for neonates [42] and larval movement increases predation risk [43], increased expression of *pin2* at the oviposition sites might contribute to elevated predation risk of neonates. Assessment of the movement of neonates on oviposition-treated tomato plants will provide a more detailed understanding of uneven expression of *pin2* around the oviposition site.

Our results suggest that *pin2* expression coincided with the emergence of neonates (Figure. 3). Although *pin2* is considered one of late response genes induced between 4 to 24 hr following herbivory [36], the observed *pin2* expression three days after oviposition is well beyond the ordinary time frame of *pin2* expression induced by mechanical wounding or insect herbivory. This delayed culmination of defense suggests that the induction of defense may be synchronized to the time of emergence of neonates. In this way, plants may be able to produce defensive compounds without wasting resources by premature expression of defense traits. Plants might be able to trace air temperature, which is most important for hatching time [44], [45], or perceive egg-derived HAMPs that indicate larvae are about to emerge. Synchronicity of defense gene induction following insect oviposition with larval emergence was recently reported [32]. The transcription of sesquiterpene synthase genes of Scots pine (*P. sylvestris* L.) was found to be the most intense 14 d following pine sawfly (*D. pini* L.) oviposition on pine branches, just prior to emergence of pine sawfly larvae.

Notably, unfertilized eggs also induced tomato *pin2* expression (Figure 4). Many females of insects fail to mate in the field [38]. A large portion of *H. zea* females were also found unmated even after spending two nights individually with a male, and virgin female moths laid unfertilized eggs for unknown reasons [39]. Brussels sprouts (*Brassica oleracea* L. var. *gemmifera*) respond to an antiaphrodisiac of its herbivorous butterfly, *P. brassicae* [46] and this compound is delivered from males to females with seminal fluid during copulation and reduces the interest of females in further mating [47]. Brussels sprouts may recognize insect oviposition through detection of this compound on leaves and may be able to even distinguish between fertilized and unfertilized eggs to save resources. However, our results indicate that tomato plants respond to eggs irrespective of egg fertility. It is interesting that tomato responded to infertile eggs, which would not lead to any feeding damage on the host plant in the future. We conjecture that in the interactions between tomato and *H. zea*, unfertilized eggs laid together with fertilized eggs might increase the “alertness” on the host plant. This is the first report of the induction of plant defensive response by deposition of unfertilized eggs.

Our results indicate that insect oviposition can prime plant defenses (Figures 5, 6). Generally, induced defense is considered more advantageous with priming [8]. Priming may reduce the possibility of development of a strategy to suppress plant defensive traits by herbivorous arthropods [48], and the cost of priming is considered relatively low [49]. Priming by oviposition should benefit plants with induction of more powerful defense upon anticipated herbivory as well as with minimized waste of resources if eggs fail to hatch or if they are removed by predators. Due to the advantages of priming and the frequency of oviposition by herbivorous insects on the host plant in the field [20], priming of defenses by insect oviposition might be a common but overlooked defense strategy of plants against future herbivory by neonates. Indeed, suppression of antiherbivore defenses by insect oviposition found recently in *Arabidopsis* [31] might be a counterploy by insects against this defensive strategy induced by insect oviposition. Interestingly, priming of plant defenses by insect oviposition was predicted [9].

In summary, we presented a series of results that indicate eggs deposited on tomato foliage by adult *H. zea* moths elicited a suite of defensive responses, including accumulation of H_2_O_2,_ expression of *pin2*, a defense gene aiming actively feeding insects, and elevated levels of the defense hormone JA. Moreover, the spatial and temporal patterns of *pin2* expression at the oviposition site were also determined. Our results indicate that oviposition primed plant defense for impending herbivory. Taken together, the results presented here suggest that, upon *H. zea* oviposition, tomato plants perceive insect eggs and induce defense directed towards larvae that will soon hatch and inflict damage on plant tissue. A former study showed egg-induced plant effects on larval performance, but did not detect the chemical or molecular causes of these effects [32]; in contrast, the present study detected egg-induced changes of JA-levels and transcript levels of a plant defense gene, but did not yet prove that these changes affect herbivore performance. In the future, it will be valuable to examine whether induction of defenses targeting neonates by insect oviposition is common in the field and how effective oviposition-induced defenses are. Characterization of potential elicitors of plant defenses may be useful for pest control as well as understanding of molecular mechanisms of oviposition-induced defense.

## Materials and Methods

### Plants and Insects

Seeds of tomato (*Solanum lycopersicum* L. cv. Better Boy) were purchased commercially. Plants were fertilized once with Osmocote Plus (15-9-12, Scotts, Marysville, OH, USA) 7–10 days after seedlings were transferred to individual pots with Pro-Mix potting soil (Premier Horticulture, Quakertown, PA, USA). Plants were grown in the greenhouse at the Pennsylvania State University (University Park, PA) on a cycle of 16-h day: 8-h night at 24–28°C. Tomato plants between the 4- to 5-leaf stages were used for oviposition treatment.

Eggs, larvae, and adults of *H. zea* were kept in an incubator on a cycle of 16-h day: 8-h night at 26°C. The eggs of *H. zea* were supplied from BioServ (Frenchtown, NJ, USA), and larvae were reared on artificial diet [50] in a 30-mL diet cup. The ingredients of artificial diet were purchased from BioServ (Frenchtown, NJ, USA) and Sigma-Aldrich (St. Louis, MO, USA). After pupation, each pupa was transferred to a new diet cup until the emergence of adults.

### Oviposition Treatment

Five to six tomato plants were caged with 20–30 females and 10–15 males of 1–3 day old *H. zea* moths in a cage (W×L×H = 75×63×88 cm) for 1.5–2 days with two scotophases in the experiments for Figures 1, 2, 5, and 6. Each moth in a cup was provided with several squirts of 10% sugar solution for 2–4 hr. Moths laid different numbers of eggs per plant from dozens to hundreds, and plants with at least 5 eggs on the distal leaflet of the 4^th^ compound leaf were used for further treatments.

In the experiment for Figure 3 where *pin2* expression at the oviposition site was traced for 3 days, moths were kept in a mating jar for 24 hr with 10% sugar solution on the bottom and squirted on the wall before they were released into cages with tomato plants inside in order to reduce the time of oviposition treatment to 1 day. In the experiment for Figure 4 to see the effect of mating on the *pin2* expression, three groups of moths of 30 virgin females, 20 virgin females with 10 virgin males, and 30 virgin males, were kept in separate mating jars with sugar solution as stated above for 24 hr, then each group of moths was released into a cage with 6 tomato plants inside.

### H_2_O_2_ Detection by DAB Staining

*H.zea* oviposition-treated tomato leaves were excised and the petioles had been dipped overnight in 1 mg mL^−1^ solution (pH 3.8) of 3,3′-diaminobenzidine (DAB) under light at the room temperature. Then, chlorophyll of leaves was removed in double-boiling ethanol and H_2_O_2_ production was visualized as brown spots. Leaves were photographed before and after dechlorophyllization [33].

### Collection of Leaf Tissue

Each leaf tissue sample was collected from an individual plant. In the experiments where *pin2* expression was measured at the oviposition site (results for Figures 1b, 2, 3, and 4), 15–20 egg-laid leaf disks of 5-mm diameter were punched off, eggs on leaf disks were removed, leaf disks were put in a 2-mL tube with a metal milling ball, frozen in liquid nitrogen, and stored at −80°C until RNA extraction. Leaf disks were sampled from the distal leaflet, and if necessary also from the medial and proximal leaflets, of the 4^th^ compound leaf. For priming tests with *pin2* (Figures 5 and S1), 50–100 mg of leaf tissue from the distal leaflet of the 4^th^ compound leaf was taken after eggs were removed, frozen with a milling ball in liquid nitrogen, and stored at −80°C until RNA extraction.

### RNA Extraction and Quantitative Real-Time Polymerase Chain Reaction (qRT-PCR)

RNA extraction was executed as previously described [18]. Leaf tissue sampled as described above was powdered with a metal milling ball in a 2 mL sample tube using GenoGrinder 2000 (Spex SamplePrep, Metuchen, NJ, USA) at 1200 strokes per min, and RNA was extracted with an RNeasy Plus Mini-kit (Qiagen, Valencia, CA, USA) following the manufacturer's instruction. cDNA was synthesized from 1 mg of RNA with High Capacity cDNA Reverse Transcription Kit (Applied Biosystems, Foster City, CA, USA) and was used as template for qRT-PCR after 10 times dilution. The sequences of the forward and reverse primers of *pin2* (Gene Bank Accession number K03291) for qRT-PCR were 5′-GGA TTT AGC GGA CTT CCT TCT G- 3′ and 5′- ATG CCA AGG CTT GTA CTA GAG AAT G- 3′, respectively. PCR product was amplified with Power SYBR Green PCR Master Mix (Applied Biosystems, Foster City, CA, USA) and the relative expression of *pin2* was analyzed with 7500 Fast Real-Time PCR System (Applied Biosystems, Foster City, CA, USA). Tomato ubiquitin gene was used as a reference gene (Gene Bank Accession number X58253) and the sequences of the forward and reverse primers were 5′-GCC AAG ATC CAG GAC AAG GA-3′ and 5′-GCT GCT TTC CGG CGA AA-3′, respectively [51].

### Test of priming by oviposition and trichome disruption

To test whether expression of tomato *pin2* is primed by *H. zea* oviposition (Figure S1), the terminal leaflet of the 4^th^ compound leaf of tomato plants treated with *H. zea* oviposition was damaged by rolling a pattern wheel 25-mm long twice paralleled with the mid vein, and 20 µL of 5-time diluted OS collected fresh from the 5^th^ instar larvae of *H. zea* was applied on the wound immediately. Leaf tissue was collected 0, 3, 8, and 24 hr after wounding treatment for RNA extraction and qRT-PCR.

To test the possibility of priming by trichome disruption, a distal leaflet of the 4^th^ compound leaf was gently rubbed with a latex-gloved finger to break down leaf glandular trichomes. Twenty four hr after disruption of trichomes, the leaflet was wounded and applied with 5 µL of *H. zea* OS to mimic *H. zea* herbivory as described above. Leaf tissue was sampled after another 24 hr for RNA and qRT-PCR. The level of *pin2* transcription was compared among intact plants, trichome-disrupted plants, wounded plants, and plants treated with both trichome breakdown and herbivory mimicry.

### Quantification of JA

The amount of JA was quantified based on the method described by Tooker and De Moraes [52]. After *H. zea* oviposition and wounding treatment and the eggs were gently removed, 100 mg of leaf tissue was sampled under liquid nitrogen into a FastPrep tubes (Qbiogene, Carlsbad, CA, USA) containing 1 g of Zirmil beads (1.1 mm; Saint-Gobain ZirPro, Mountainside, NJ, USA), 400 µL of extraction buffer (1-PrOH∶H_2_O∶HCl = 2∶1∶0.002, v/v), and 100 ng of dihydrojasmonic acid (diH-JA) as an internal standard. DiH-JA was obtained by alkaline hydrolysis of methyl dihydrojasmonate (Bedoukian Research Inc., Danbury, CT, USA). Leaf tissue was sampled 0, 30, 60, and 180 min after treatment with wounding and *H. zea* OS treatment and stored at −80°C until necessary.

Plant leaf tissue was shredded in FastPrep FP120 (ThermoSavant, Holbrook, NY) for 40 sec at 5.5 unit speed at the room temperature. After 1 mL of CH_2_Cl_2_ was added, FastPrep tubes were shaken against in FastPrep FP120 for 40 sec at 5.5 unit speed at the room temperature. After centrifugation at 10,000 *g* for 1 min (Heraeus Biofuge Pico, Thermo Fisher Scientific, Waltham, MA), the organic layer was transferred to a 4-mL screw-capped glass vial with a glass syringe (Hamilton Company, Reno, NV) and dried up under gentle air flow at the room temperature. JA in the dried samples were methylesterificated into methyl jasmonate (MJ) with 2.3 µL of trimethylsilyl diazomethane (TMS-CH_2_N_2_; 2M in hexane; Sigma-Aldrich, St. Louis, MO, USA) in 100 µl of MeOH/diethyl ether (1∶9, v/v) for 25 min at the room temperature. The remaining TMS-CH_2_N_2_ was neutralized by addition of 2.3 µL of hexane/AcOH (88∶12, v/v) for additional 25 min at the room temperature. MJ was evaporated at 200°C into a SuperQ (80/100 mesh; Alltech, Deerfield, IL) trap for 2 min and recovered with 150 µL of CH_2_Cl_2_ into a glass insert in a GC vial for GC-MS analysis.

MJ was chemically ionized with isobutene and analyzed on the selected ion monitoring mode by GC/MS (6890 Plus/5973N, Agilent, Santa Clara, CA) equipped with HP-1MS column (length 30 m, inner diameter 0.25 mm, film thickness 0.25 µm; Agilent, Santa Clara, CA). The injection port was maintained at 250°C and the oven temperature was kept on 40°C for 1 min, increased by the rate of 15°C min^−1^, and maintained at 250°C for 7 min.

### Statistics

All the data were subject to Grubb's test to statistically remove outliers (*p*<0.05; Graphpad Software). When log transformed data satisfied the assumptions of normality and equal variances, significant difference of data was determined with Proc GLM, and when the assumptions were not satisfied, non-parametric GLM was used instead. Multiple comparison of data was carried out with Tukey test (SAS 9.3, SAS Inc.).

## Supporting Information

Figure S1 **Effect of trichome disruption on the level of** ***pin2*** **expression upon subsequent mechanical wounding and applicaton of** ***H. zea*** **OS.** Trichome disruption did not influence the level of *pin2* expression upon subsequent simulated herbivory. Data were analyzed for significance with non-parametric Proc GLM and compared with Tukey test (Mean ± SE; Chi-Square = 17.3945, *p* = 0.006, *N* = 5 or 6; letters above bars indicate significant difference).
(DOCX)
