# Supplementary material for: Insect Eggs Can Enhance Wound Response in Plants: A Study System of Tomato Solanum lycopersicum L. and Helicoverpa zea Boddie
Source: PLoS One. 2012 May 15;7(5):e37420. doi: 10.1371/journal.pone.0037420 (PMC3352884; doi:10.1371/journal.pone.0037420)
Supplement: Supplementary file 1 [file pone.0037420.s001.docx]

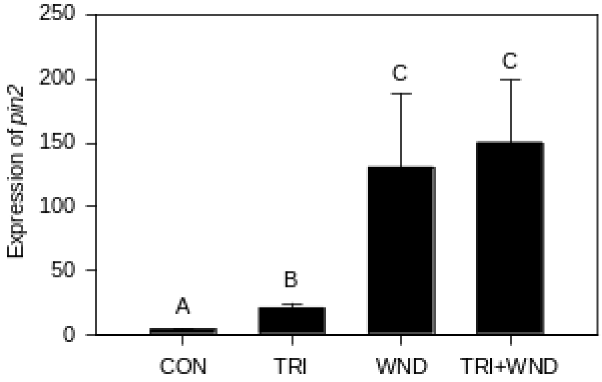
Expression of pin2
250
200
150
100
50
0
A
B
C
C
CON
TRI
WND
TRI+WND
